# A Prospective Multicenter Registry on Feasibility, Safety, and Outcome of Endovascular Recanalization in Childhood Stroke (Save ChildS Pro)

**DOI:** 10.3389/fneur.2021.736092

**Published:** 2021-09-03

**Authors:** Peter B. Sporns, André Kemmling, Sarah Lee, Heather Fullerton, Wolfgang G. Kunz, Jenny L. Wilson, Mark T. Mackay, Maja Steinlin, Jens Fiehler, Marios Psychogios, Moritz Wildgruber

**Affiliations:** ^1^Department of Neuroradiology, Clinic of Radiology and Nuclear Medicine, University Hospital Basel, Basel, Switzerland; ^2^Department of Diagnostic and Interventional Neuroradiology, University Medical Center Hamburg-Eppendorf, Hamburg, Germany; ^3^Department of Neuroradiology, University Hospital Marburg, Marburg, Germany; ^4^Division of Child Neurology, Department of Neurology, Stanford University, Stanford, CA, United States; ^5^Department of Neurology and Pediatrics, University of California, San Francisco, San Francisco, CA, United States; ^6^Department of Radiology, University Hospital, Munich, Germany; ^7^Division of Pediatric Neurology, Oregon Health & Science University, Portland, OR, United States; ^8^Department of Neurology, The Royal Children's Hospital, Melbourne, VIC, Australia; ^9^Division of Neuropediatrics, Development and Rehabilitation, Children's University Hospital, Inselspital, Bern University Hospital, University of Bern, Bern, Switzerland

**Keywords:** stroke, ischemic stroke, arterial ischemic stroke, thrombectomy, mechanical thrombectomy, intravenous thrombolysis, childhood stroke, pediatric stroke

## Abstract

**Rationale:** Early evidence for the benefit of mechanical thrombectomy (MT) in pediatric patients with intracranial large vessel occlusion has been shown in previous retrospective cohorts. Higher-level evidence is needed to overcome the limitations of these studies such as the lack of a control group and the retrospective design. Randomized trials will very likely not be feasible, and several open questions remain, for example, the impact of arteriopathic etiologies or a possible lower age limit for MT. Save ChildS Pro therefore aims to demonstrate the safety and effectiveness of MT in pediatric patients compared to the best medical management and intravenous thrombolysis.

**Design:** Save ChildS Pro is designed as a worldwide multicenter prospective registry comparing the safety and effectiveness of MT to the best medical care alone in the treatment of pediatric arterial ischemic stroke (AIS). It will include pediatric patients (<18 years) with symptomatic acute intracranial arterial occlusion who underwent either MT or best medical treatment including intravenous thrombolysis.

**Outcomes:** The primary endpoint of Save ChildS Pro is the modified Rankin Scale score at 90 days post-stroke. Secondary endpoints will comprise the decrease of the Pediatric National Institutes of Health Stroke Scale score from admission to discharge and rate of complications.

**Discussion:** Save ChildS Pro aims to provide high-level evidence for MT for pediatric patients with AIS, thereby improving functional outcome and quality of life and reducing the individual, societal, and economic burden of death and disability resulting from pediatric stroke.

**Clinical Trial Registration:** Save ChildS Pro is registered at the German Clinical Trials Registry (DRKS; identifier: DRKS00018960).

## Introduction

Arterial ischemic stroke (AIS) affects 1.3 to 1.6 per 100,000 children every year in developed countries (1–3), and outcomes are potentially severe, with 70% retaining long-term neurological deficits, 20% suffering recurrent strokes, and 10% of strokes resulting in death of the child (4–6). Risk factors for adult stroke such as atherosclerosis are nearly non-existent in pediatric stroke, so that knowledge and evidence of adult stroke cannot be extrapolated to children. Moreover, a lack of recruitment led to the termination of the prospective randomized TIPS (Thrombolysis in Pediatric Stroke) trial designed to assess the safety of intravenous tissue-type plasminogen activator (iv-tPA) in pediatric patients, highlighting the challenges of randomizing children with acute stroke (7). For mechanical thrombectomy (MT) in adults, several randomized clinical trials published in 2015 have shown the efficacy and safety of endovascular recanalization for large vessel occlusions (LVOs) with large effect size (8). In children, after several case series (9, 10), the retrospective Save ChildS Study recently provided the first systematic evidence for the safety of MT in children (11–13). In this study, the rate of recanalization and adverse events was comparable to large randomized controlled trials in adults, and neurological outcomes of the children were generally favorable. However, definitive conclusions cannot be drawn given the limitations of the Save ChildS Study, which include the lack of a control group of LVO patients not treated by MT and the retrospective design (14). Important questions remain, such as a possible lower age limit to perform thrombectomy in children or the impact of specific etiologies such as cerebral arteriopathies on the safety and efficacy of MT.

With the abovementioned difficulties in recruiting pediatric stroke patients for randomized controlled trials together with the ethical concerns about randomizing children into a non-MT group on the basis of the current knowledge, prospective randomized trials on MT in children are unlikely to succeed. Therefore, a prospective multicenter registry is considered the best option to collect further evidence for performing interventional recanalization in childhood stroke (14, 15).

The primary objective of the Save ChildS Pro registry is therefore to generate evidence for the use of MT in childhood stroke under the hypothesis that MT is safe and results in a high rate of good clinical outcomes compared to the best medical treatment including intravenous thrombolysis. The Save ChildS Pro registry further serves the purpose of defining selection criteria for MT in pediatric patients, especially for potentially vulnerable subgroups such as very young children, children with AIS due to arteriopathy, and children in the late therapeutic window.

## Methods

### Design

Save ChildS Pro is a worldwide multicenter prospective registry that aims to compare the safety and effectiveness of MT to the best medical care alone (including intravenous thrombolysis) in the treatment of pediatric AIS. As of January 2020, 50 centers in nine countries (Germany, Switzerland, Austria, Italy, Sweden, United States, Australia, Argentina, Canada) have agreed to participate. Patient data will be entered through a web-based interface (Eppdata).

Save ChildS Pro is registered at the German Clinical Trials Registry (DRKS; identifier DRKS00018960) and has been approved by the ethics committee of the University of Muenster, Muenster, Germany, in accordance with the Declaration of Helsinki, with waiver of informed consent due to anonymization of the submitted data.

### Patient Population

Patients presenting with AIS from January 1, 2020, will be considered for prospective study enrolment.

The following inclusion criteria apply:

- clinical diagnosis of acute ischemic stroke
- confirmed diagnosis of arterial occlusion consistent with symptoms, including occlusion of terminal carotid artery, middle cerebral artery (M1, M2, M3 segments), basilar artery, anterior cerebral artery (A1, A2 segments), or posterior cerebral artery (P1, P2 segments)
- endovascular treatment (EVT) attempted (i.e., groin puncture initiated, including all cases where EVT failed or was interrupted) or best medical treatment including intravenous thrombolysis
- age <18 years

The following is the exclusion criterion:

- perinatal stroke

### Study Groups

#### Endovascular Group

Patients treated with MT (i.e., groin puncture initiated, including all cases where EVT failed or was interrupted), according to established clinical practice, belong to the endovascular group.

#### Best Medical Treatment Group

The best medical treatment group includes patients who are treated with best medical therapy (including systemic thrombolysis), according to established clinical practice.

## Outcomes

### Primary Outcomes

The primary endpoint of Save ChildS Pro is the patient's modified Rankin Scale score (mRS) at 90 days post-stroke assessed with a shift analysis.

### Secondary Outcomes

Secondary endpoints will comprise the decrease of the Pediatric National Institutes of Health Stroke Scale (PedNIHSS) score from admission to discharge and rate of complications. The study will undergo an interim analysis after the first 50 patients have been enrolled.

#### Clinical and Radiological Assessment

The following baseline and disease characteristics will be assessed at the timepoint admission, treatment, 24 h after treatment, discharge from hospital, and at day 90 (±10 days).

##### Admission

- Patient data (age, year of birth, gender)
- Patient logistics (symptom onset, admission weekday, date and time of admission, date and time of admission imaging, date and time of last known well, date, and time of symptom onset, referral from other hospital)
- Pre-existing diseases
- Medication
- mRS at admission (deficits prior to stroke)
- PedNIHSS
- Imaging findings (type of admission imaging, occluded vessel, computed tomography or magnetic resonance imaging for Alberta stroke program early computed tomography (ASPECT) score, ASPECT score)

##### Treatment

- Treatment with iv-tPA

- date and time of iv-tPA
- dose

- EVT

- timing (date and time of first angiography image, time of first pass, time of final recanalization result)
- type of anesthesia
- occluded vessel
- stenosis or occlusion in a proximal vessel
- number of passes
- morphologic appearance (normal, arteriopathy, other)
- type of treatment and devices used,
- treatment complications [vasospasm, intracerebral hemorrhage (ICH), dissection, other]

##### 24 h

- PedNIHSS
- Imaging findings (persistent or new occlusion, type of follow-up imaging, ASPECTS)
- Adverse events (symptomatic ICH, non-symptomatic ICH, dissection, other)

##### Discharge

- Patient logistics (length of stay, transfer destination)
- PedNIHSS
- mRS
- Pediatric Stroke Outcome Measure (PSOM)
- Stroke etiology (according to the childhood arterial ischemic stroke standardized classification and diagnostic evaluation classification)
- Adverse events (symptomatic ICH, non-symptomatic ICH, hemicraniectomy, external ventricular drainage, other)

##### Day 90 ± 10

- mRS
- PSOM
- Adverse events (symptomatic ICH, non-symptomatic ICH, hemicraniectomy, external ventricular drainage, pneumonia, recurrent stroke, other)
- Location (care facility, home)

#### Data Collection

Data will be collected as part of routine clinical care.

#### Funding

Eppdata will provide data services for free in the beginning; later on, acquisition of funding is planned.

#### Data Ownership

Each participating center remains owner of its data. Upon request, data can be retracted from the database.

#### Publication Policy

Inclusive (multiauthor): those doing the work will receive the credit, every center that contributes a relevant number of cases will have the right of coauthorship in publications.

#### Number of Sites

Not limited.

#### Study Design

Non-interventional, open-label, prospective, multicenter, observational registry study.

### Statistical Analyses

Summary tables for patient demographics and baseline characteristics will be provided, and comparisons will be made between study arms for the endovascular and best medical treatment groups. The primary and secondary outcomes will be summarized and compared between study groups for the endovascular and best medical treatment groups.

In general, summaries will be presented by occlusion location within treatment group in the relevant analysis populations. Descriptive statistics for dichotomous/categorical variables will include the number and percent of subjects in each category (including missing), by treatment group. Descriptive statistics for continuous variables will include the number of non-missing and missing variables, minimum, lower quartile, median, upper quartile, maximum, mean, and standard deviation, stratified by treatment group. Regarding comparisons between treatment groups, χ^2^ test (Fisher exact test where appropriate) will be utilized for the comparison of categorical variables, and the *t* test (or the Mann–Whitney *U*-test when appropriate) will be utilized for the comparison of continuous variables. Data for the primary outcome will be presented by treatment group. Confidence intervals for dichotomous or ordinal endpoints will be reported on the odds ratio scale.

## Conclusion

Save ChildS Pro is a worldwide multicenter prospective registry to compare the safety and effectiveness of endovascular thrombectomy to the best medical care alone (including intravenous thrombolysis) in the treatment of pediatric AIS. Save ChildS Pro will provide high-level evidence regarding effectiveness and safety of thrombectomy for pediatric AIS, improving the ability to determine which children are likely to benefit from this therapy.

## Ethics Statement

The studies involving human participants were reviewed and approved by University of Münster. Written informed consent from the participants' legal guardian/next of kin was not required to participate in this study in accordance with the national legislation and the institutional requirements.

## Author Contributions

PS and MW contributed to conceptualization and design. All authors drafted the manuscript, critically revised it and collect the data.

## Conflict of Interest

The authors declare that the research was conducted in the absence of any commercial or financial relationships that could be construed as a potential conflict of interest. The handling Editor declared a shared affiliation, though no other collaboration, with one of the authors, MS.

## Publisher's Note

All claims expressed in this article are solely those of the authors and do not necessarily represent those of their affiliated organizations, or those of the publisher, the editors and the reviewers. Any product that may be evaluated in this article, or claim that may be made by its manufacturer, is not guaranteed or endorsed by the publisher.
